# Serological Detection of SARS-CoV-2 Antibodies in Naturally-Infected Mink and Other Experimentally-Infected Animals

**DOI:** 10.3390/v13081649

**Published:** 2021-08-19

**Authors:** Francisco J. Berguido, Peter D. Burbelo, Alessio Bortolami, Francesco Bonfante, Kerstin Wernike, Donata Hoffmann, Anne Balkema-Buschmann, Martin Beer, William G. Dundon, Charles E. Lamien, Giovanni Cattoli

**Affiliations:** 1 Joint FAO/IAEA Centre for Nuclear Applications in Food and Agriculture, Animal Production and Health Laboratory, Department of Nuclear Sciences and Applications, International Atomic Energy Agency Vienna International Centre, P.O. Box 100, 1400 Vienna, Austria; w.dundon@iaea.org (W.G.D.); c.lamien@iaea.org (C.E.L.); g.cattoli@iaea.org (G.C.); 2 National Institute of Dental and Craniofacial Research, National Institutes of Health, Bethesda, MD 20892, USA; burbelop@nidcr.nih.gov; 3 Laboratory of Experimental Animal Models, Division of Comparative Biomedical Sciences, Istituto Zooprofilattico Sperimentale delle Venezie, 35020 Legnaro, Italy; ABortolami@izsvenezie.it (A.B.); FBonfante@izsvenezie.it (F.B.); 4 Institute of Diagnostic Virology, Friedrich-Loeffler-Institut, Südufer 10, 17493 Greifswald, Insel Riems, Germany; Kerstin.Wernike@fli.de (K.W.); donata.hoffmann@fli.de (D.H.); Martin.Beer@fli.de (M.B.); 5 Institute of Novel and Emerging Infectious Diseases, Friedrich-Loeffler-Institut, Südufer 10, 17493 Greifswald, Insel Riems, Germany; Anne.Balkema-Buschmann@fli.de

**Keywords:** SARS-CoV-2, mink, animal, luciferase immunoprecipitation systems, sera, nucleocapsid, spike

## Abstract

The recent emergence of SARS-CoV-2 in humans from a yet unidentified animal reservoir and the capacity of the virus to naturally infect pets, farmed animals and potentially wild animals has highlighted the need for serological surveillance tools. In this study, the luciferase immunoprecipitation systems (LIPS), employing the spike (S) and nucleocapsid proteins (N) of SARS-CoV-2, was used to examine the suitability of the assay for antibody detection in different animal species. Sera from SARS-CoV-2 naturally-infected mink (*n* = 77), SARS-CoV-2 experimentally-infected ferrets, fruit bats and hamsters and a rabbit vaccinated with a purified spike protein were examined for antibodies using the SARS-CoV-2 N and/or S proteins. From comparison with the known neutralization status of the serum samples, statistical analyses including calculation of the Spearman rank-order-correlation coefficient and Cohen’s kappa agreement were used to interpret the antibody results and diagnostic performance. The LIPS immunoassay robustly detected the presence of viral antibodies in naturally infected SARS-CoV-2 mink, experimentally infected ferrets, fruit bats and hamsters as well as in an immunized rabbit. For the SARS-CoV-2-LIPS-S assay, there was a good level of discrimination between the positive and negative samples for each of the five species tested with 100% agreement with the virus neutralization results. In contrast, the SARS-CoV-2-LIPS-N assay did not consistently differentiate between SARS-CoV-2 positive and negative sera. This study demonstrates the suitability of the SARS-CoV-2-LIPS-S assay for the sero-surveillance of SARS-CoV-2 infection in a range of animal species.

## 1. Introduction

Although the zoonotic nature of SARS-CoV-2 is generally accepted, its animal reservoir remains unidentified. Horseshoe bats (*Rhinolophus* spp.) have been reported to harbor the most genetically similar virus to SARS-CoV-2, which is believed to have passed through a second unknown animal host before being transmitted to humans [1,2]. There is concern that the currently circulating virus may transmit to a new (wild) animal reservoir, evolve to evade treatments and vaccines and become a source of recurrent infections in humans, much like wild birds and influenza A viruses [3,4]. Indeed, detection of SARS-CoV-2 RNA in the lymph nodes of two feral mink in eastern Spain has recently been reported [5]. Of additional concern is the potential for recombination events between SARS-CoV-2 and other coronaviruses in the animal host, as has been observed for other coronaviruses [6,7,8,9]. Additionally, since its emergence, there have been several reported infections (both natural or experimental) of different animal species with SARS-CoV-2 including dogs, cats, cattle, ferrets, captive gorillas, lions, tigers, pumas, snow leopards, racoon-dogs, bats, white-tailed deer and minks [2,10].

Natural SARS-CoV-2 infection of farmed mink were first reported in the Netherlands between April and May 2020 [11]. As of July 2021, SARS-CoV-2 infection has been detected in mink farms across nine additional European countries including Denmark, France, Greece, Italy, Latvia, Lithuania, Poland, Spain and Sweden and in Canada and the United States [12,13]. As a result, the European Food Safety Authority (EFSA) and the European Centre for Disease Prevention and Control (ECDC) have recommended the coordinated monitoring of mink farms in its Member States [12]. A risk assessment was also undertaken by the Joint FAO–OIE–WHO Global Early Warning System for health threats and emerging risks at the human–animal–ecosystems interface on data from 36 countries in Africa, Asia, Europe, South and North America, where animals of the families Mustelidae, Leporidae and Canidae are commercially farmed for fur. A list of recommended mitigation measures has been introduced to reduce the likelihood of SARS-CoV-2 infection in humans and to prevent its introduction and spread within fur farms [14].

Understanding SARS-CoV-2 infection in animal species and the identification of potential future animal reservoirs or intermediate hosts are crucial for a better understanding and eventual disease control. Although molecular methods are fast, reliable and highly sensitive, they only detect viral RNA from active infections. Serological assays measuring antibodies, on the other hand, can determine whether hosts have a current or had a past exposure to the virus. Besides serological assays that generally measure only antibody titer, additional insight can be obtained by studying whether serum antibodies are able to neutralize SARS-CoV-2 virus infectivity in vitro. These assays, such as virus neutralization test (VNT) and plaque reduction neutralization test (PRNT), are commonly used as reference methods to compare serological assays and for the development of vaccines [15,16].

A variety of immunological assays including indirect immunofluorescence assay (iIFA), enzyme-linked immunosorbent assays (ELISA), chemiluminescence immunoassays, and lateral flow immunochromatographic assays, are used to evaluate SARS-CoV-2 antibodies for understanding serological evidence of infection [17,18]. In addition, the fluid phase luciferase immunoprecipitation systems (LIPS) utilizing light-emitting chimeric fusion proteins of the nucleocapsid protein (N), and the spike protein (S) were used to study COVID-19 infection in humans [19,20,21]. In several of the studies using LIPS, the immunoassay showed high sensitivity and specificity for detecting SARS-CoV-2 in humans.

It should be noted that LIPS has a number of advantages over other immunoassay formats that make this approach a potentially strong candidate for sero-surveillance and monitoring of wild, laboratory and farmed animals for SARS-CoV2 infection. Firstly, the LIPS assay is performed in solution allowing for the maintenance of the native antigen conformation, which is not the case for other assays. As the assay does not use a live virus, LIPS does not require BSL3 facilities unlike iIFA and VNT. Another advantage of LIPS is that it does not require a species-specific secondary antibody, thus making it suitable for the screening of multiple species simultaneously. Lastly, LIPS requires only small volumes of sera for testing (1 µL per reaction), which is particularly important when testing small mammals (e.g., bats, rodents) or wildlife samples, where the amount of blood or other biological fluids is limited. [22]. For the reasons above, and our past experience with the technology for the identification of antibodies against the peste des petits ruminants virus in sheep and goat sera [23], we evaluated the possibility of using LIPS to detect SARS-CoV-2 antibodies in mink and other animals.

## 2. Materials and Methods

### 2.1. Serum Samples

Several different animal species were tested for SARS-CoV-2 serum antibodies (Table 1). Seventy-five serum samples from SARS-CoV-2-infected mink were collected from a farm located in the Northeast of Italy, during surveillance activities conducted between January and March 2021, as part of the national surveillance program for monitoring SARS-CoV-2 in mink farms. Blood samples from minks were collected by puncture of the cranial vena cava under anesthesia induced by a ketamine/medetomidine formulation and subsequently reversed with atipamezole. Thirty, 25 and 22 sera were collected on the first, second (+7 days) and third (+65 days) visits, respectively. The first sampling led to the identification by real-time RT-PCR of one infected animal. Two sera from pet minks with no clinical history of infection that had tested negative by virological and serological analyses were also included in the study.

In the context of a research project aimed at testing the efficacy of antiviral molecules for the treatment of COVID-19, twenty-six Golden Syrian Hamsters of approximately 120 g (8–10 weeks of age) were sampled for the collection of blood, at the Istituto Zooprofilattico Sperimentale delle Venezie, Italy. Under BSL-3 conditions, hamsters were challenged via the nasal route with either 40,000 focus forming units of SARS-CoV-2 (*n* = 22 hamsters) or a saline mock inoculum (*n* = 4 hamsters). The challenged hamsters developed mild clinical symptoms, experiencing moderate (10%) weight loss and persistent viral shedding for over 7 days post infection (p.i.) (data not shown). On day 14 p.i., all hamsters were anesthetized with a ketamine/xylazine formulation and exsanguinated via cardiac puncture.

Other sera tested in this study were from experimental infections of ferrets and *Rousettus aegyptiacus* fruit bats [24]. In addition, serum taken from a rabbit immunized twice with a purified spike protein (S1; amino acids 17 to 685) [25] was included. Negative sera were obtained from different sources as listed in Table 1. The negative or positive status of each serum was confirmed by PRNT (minks, hamsters), VNT (ferrets, bats, rabbits) and/or ELISA (ferrets, rabbits) as previously described [15,25].

### 2.2. Luciferase Immunoprecipitation Systems (LIPS)

Previously described pREN2 and pGaus3 plasmids encoding genes for the luciferase fusion proteins of SARS-CoV-2 N and S protein, respectively, were utilized [19]. The assays were performed as previously described using two separate target antigens [19,23].

Briefly, Cos 7 cells grown in DMEM supplemented with 10% fetal bovine serum were transfected using the Fugene 6 protocol (Promega, Madison, WI, USA) with plasmids pREN2 SARS-CoV-2-Nucleocapsid for the SARS-CoV-2-LIPS-N assay (referred to as LIPS-N from now on) or pGaus3 SARS- CoV-2-Spike for the SARS-CoV-2-LIPS-S (referred to as LIPS-S from now on). Two days after transfection, medium was removed and the cells were washed with 6 mL of phosphate buffered saline (PBS), followed by the addition of 1.4 mL of cold lysis buffer (50 mM Tris, pH 7.5, 100 mM NaCl, 5 mM MgCl_2_, 1% Triton X-100, 50% glycerol and protease inhibitor (2 tablets of complete protease inhibitor cocktail (Roche, Mannheim Germany) per 50 mL of lysis buffer). Cells were then harvested and dissociated using a sonicator (Vibra-Cell VCX 750, Sonics and Materials Inc., Newtown, CT, USA). The samples were centrifuged at 16,000× *g* for 4 min at 4 °C and the supernatants were collected and stored at −80 °C until required. To determine total luciferase activity, 1 μL of crude fusion protein extract was added to 100 μL of coelenterazine substrate (Promega, Madison, WI, USA) in a white 96 well-plate (Sterilin, Thermo Fisher Scientific, Waltham, MA, USA). Relative light units (RLU) were measured in a luminometer (Berthold Centro LB, Berthold Technologies, Bad Wildbad, Germany) for 5 s and the volume of protein extract required to produce 1 × 10^7^ RLU was determined.

LIPS assays for measuring antibodies in the serum samples were carried out by mixing 40 μL of buffer A (50 mM Tris, pH 7.5, 100 mM MgCl_2_, 1% Triton X-100), 10 μL of diluted serum (1 in 10 in buffer A) and 50 μL of buffer A containing enough fusion protein extract to generate 1 × 10^7^ RLU (as calculated above) in each well of a 96-well plate. This mixture was incubated for 1 h at room temperature with gentle shaking. The mixture was then transferred to a 96 well Multi-Screen HTS filter plate (Millipore) and incubated with 5 μL of Ultralink immobilized protein A/G beads (Pierce Biotechnology, Thermo Fisher Scientific, Waltham, MA, USA) for 1 h at room temperature with gentle shaking, and then washed 8 times with buffer A and twice with PBS using a vacuum manifold. Coelenterazine substrate was added and the light emission was read for 5 s using a luminometer (Berthold Centro LB, Berthold Technologies, Bad Wildbad, Germany). Samples were tested in duplicate on two separate days for a total of four replicates. 

### 2.3. Statistical Analysis

For each sample, the mean value of the RLUs from two independent runs were used. Statistical analysis and data representation were carried out using the R statistical package. Wilcoxon and Dunn’s tests were used to compare the positive and negative values obtained for each species. Box plots were used to visualize the samples per group of the positive and negative population by species. 

The correlation between the PRNT, the LIPS-S and LIPS-N assay was analyzed using Spearman correlation coefficient. In addition, the LIPS results were converted to categorical data (i.e., positive or negative) for agreement analysis by the Fleiss kappa test to assess the reliability of the overall agreement between the three methods (LIPS-S, LIPS-N and PRNT) using the Interrater Reliability (irr) package in R. The agreement between pairs of assays (i.e., LIPS-S vs. LIPS-N, LIPS-S vs. PRNT; LIPS-N vs. PRNT) was determined through the Cohen’s kappa coefficient (κ) and confidence bands, using the Visualizing Categorical Data (vcd) package in R. All plots were produced using the ggplot2 package.

## 3. Results

### LIPS Assays Evaluation Using Different Animal Sera

LIPS assays using the SARS-CoV-2 spike (LIPS-S) and nucleocapsid (LIPS-N) fusion proteins were evaluated against a panel of sera from different animal species. Sera from ferret, fruit bat, hamster, mink and rabbit were tested by the LIPS-S assay, while only ferret, hamster and mink sera were tested by the LIPS-N assay due to limited availability of bat sera and the fact that the rabbit was immunized with only the SARS-CoV-2 S antigen [26].

**Figure 1:**
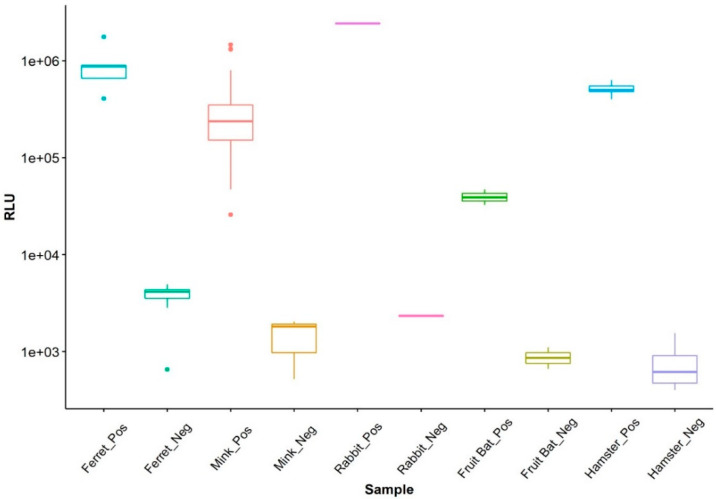
Boxplot showing the differences between the LIPS-S negative and positive samples and the relative similar levels of antibodies found in the negative samples across species.

**Figure 2:**
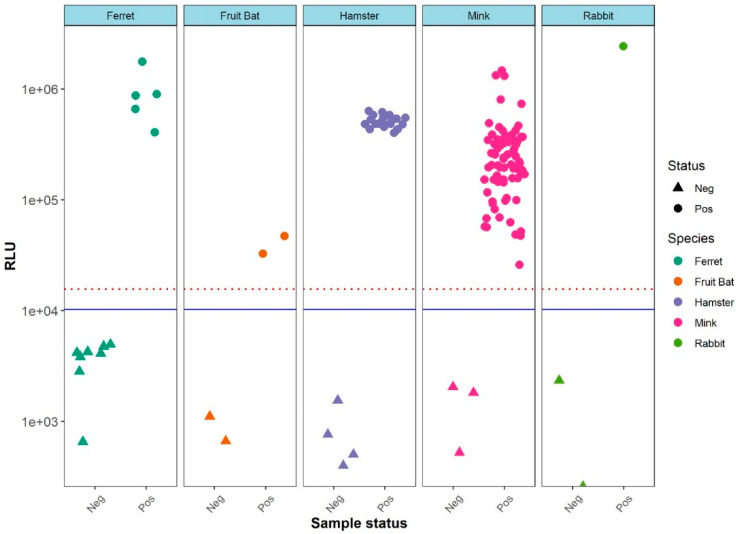
Distribution of the LIPS-S assay antibody values based of the sample’s known SARS-CoV-2 antibody neutralization status. The RLU determined by LIPS is shown on the *Y*-axis. Of note, all the negative samples are located below the blue threshold line of mean plus three standard deviations. A threshold line of mean plus five standard deviations (red dotted line) is also included for reference.

For the LIPS-S assay, there was a good discrimination between the positive and negative samples for each of the five species tested (Figure 1). Pairwise comparison showed that the mean values of the positive samples were significantly higher than those of the negative samples of the same species (*p* < 0.05 for all pairs). Ferret sera displayed a higher variation of values for negative samples; however, the analysis of variance showed that the negative sample values did not differ significantly between species (*p* > 0.05). Hence, to compensate for the low number of negative samples in some of the species under consideration (i.e., rabbit and fruit bat), a cut-off for the LIPS-S assay based on the negative values for all species was calculated, based on the mean plus three standard deviations, giving a cross- species cut-off. Using the cross-species cut-off, a clear separation of positive and negative sera was evident in all five species (Figure 2). This classification of sera as LIPS-S positive or negative was in good agreement with neutralization activity (i.e., either PRNT or VNT) results for each of the five animal species (Figure 2). 

**Figure 3:**
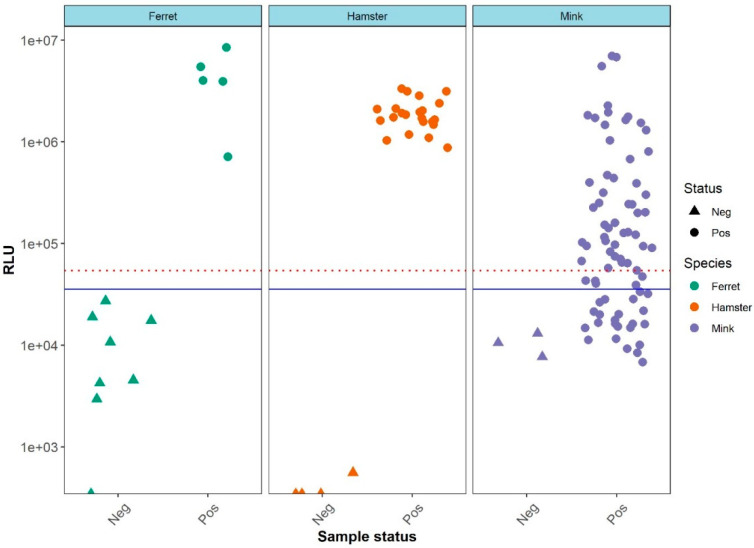
Distribution of the LIPS-N assay RLU values based of the sample’s SARS-CoV-2 antibody neutralization Note that all true negative samples are located below the blue threshold line of mean plus three standard deviations. A threshold line of mean plus five standard deviations (red dotted line) is also included for reference.

For the LIPS-N assay a similar cross-species cut-off value was calculated. In contrast to the LIPS-S assay, the separation of positive and negative sera using the LIPS-N assay (Figure S1) was only statistically significant (*p* < 0.05) for the ferret and hamster sera. This observation was reflected in the classification of sera as positive or negative by the LIPS-N assay compared to their neutralization test (NT) status (Figure 3). The distribution of the RLUs generated by the mink sera as tested by the LIPS-N assay was clearly not separated according to their NT status (Figure 3).

**Figure 4:**
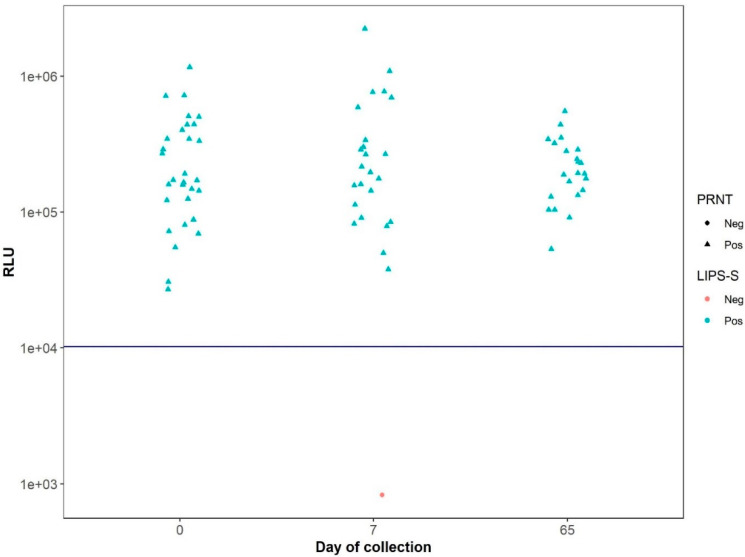
Distribution of RLU values determined by LIPS-S assay using sera from naturally-infected mink samples across the lower threshold line (blue) and according to the serum collection date. Note that, independent of the serum collection times, the LIPS-S assay correctly identified all samples according to their positive and negative status as determine by antibody neutralization activity.

**Figure 5:**
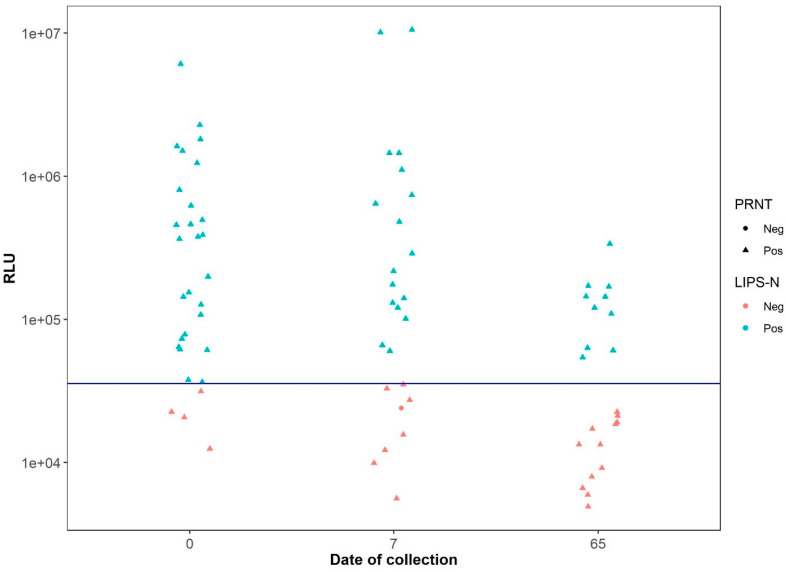
Distribution of RLU values generated using the sera from naturally SARS-CoV-2-infected mink samples across the threshold line for the LIPS-N assay according to the serum collection date. Note that the RLU values generated from NT positive sera declined over time using LIPS-N and an increased number of NT positive sera fall below the threshold line.

Because the mink sera panel was the largest panel (*n* = 77) tested and were the only sera collected from animals that had been naturally infected by SARS-CoV-2, further analyses of these sera were performed. When analyzing the LIPS-S assay data according to the collection date, it was observed that there was a consistent and accurate separation of the negative and positive samples in perfect agreement with the NT results (Figure 4). In contrast, the data from the LIPS-N assay showed that the RLU values for a number of the samples deemed positive by neutralization test declined between the first and third sampling periods (Figure 5).

Further correlation (Spearman) and agreement (Fleiss kappa and Cohen kappa) analyses were carried out using the antibody data obtained for the mink sera in both LIPS assays with the corresponding neutralization activity. Overall, a significantly strong positive correlation (r = 0.74) between RLU values of the LIPS-S assay and neutralization titers was observed which was independent of the sample collection periods (although a slight reduction in correlation was seen for the third sampling period) (Table S1). Similarly, there was a significant to moderately positive correlation (r = 0.65) between the LIPS-N RLU values and neutralization status (Table S1). The Fleiss kappa was calculated as 0.00319 suggesting a poor overall agreement between the three methods, while the Cohen kappa agreement analysis revealed a 100% agreement between the LIPS-S assay and neutralization status independently of when the samples were collected (Table S2). In contrast, there was only a weak-to-moderate agreement between the LIPS-N and both the NT and the LIPS-S status (positive or negative) of the sera samples. When the mink samples were analyzed based on the collection date, the lowest agreement (Cohen kappa = 0.122) between the LIPS-N assay and each of the two other methods (i.e., LIPS-S and PRNT) was from the sera collected during the last collection period (i.e., day 65), suggesting a decline in the anti-nucleocapsid antibodies in the sera at that sampling time point.

## 4. Discussion

Given the evidence for susceptibility of wild, domestic and farmed animals to infection by SARS-CoV-2 and the public health risks associated with these infections, there is an urgent need for suitable tools for the sero surveillance and monitoring of different animal species. To date, there are just a few serological testing methods for SARS-CoV-2 in animals based on either ELISA, PRNT, VNT or an indirect immunofluorescence assay [26,27,28,29,30]. One study has employed LIPS using both the S and N target to screen a small number of dogs (*n* = 12) and cats (*n* = 9) that had repeated contacts with SARS-CoV-2-infected humans, but the authors did not detect any anti-SARS-CoV-2 antibodies in the sera of these animals [31].

Presently, there are no reports on validation studies of serological assays used to detect antibodies to SARS-CoV-2 in minks, a limitation that has been recently highlighted by EFSA [12]. Our study indicates that LIPS is a suitable immunoassay for serological surveys in mink. Using this assay, it was clearly possible to distinguish between positive and negative animals, particularly when the spike protein was used as a target antigen. The LIPS-S also showed good diagnostic sensitivity and was capable of detecting positive sera that has a PRNT titer as low as 1:10. Despite these promising results, further work is required to fully validate the LIPS assay which should include repeatability, analytical and diagnostic sensitivity and specificity determinations. In addition, because of the low number of sera, particularly negative control sera for each animal species, future evaluation studies will require larger numbers of well-defined sera.

In humans it has been reported that the SARS-CoV-2 anti-N antibody responses wane more rapidly post-infection as compared to the anti-S response, which persist over time [32]. From our study it seems that a similar phenomenon may be occurring in naturally infected mink. It is known from experimental studies in ferrets that SARS-CoV-2 RNA can be detected between 2 and 14 days post-infection, while antibodies can be detected from eight to at least 36 days post infection [33]. In mink, seroconversion have been detected from 18 days post infection following experimental inoculation with SARS-CoV-2, however the duration of detectable antibodies was not reported [34]. Although we cannot determine the exact time at which the minks in the monitored farm were naturally infected, or whether there were multiple introductions and/or infections, the fact that there was only one PCR-positive animal detected during the first visits to the farm (day 0) suggests that the animals were first sampled at least 14 days after the infection. This would mean that, on the third sampling, some of the animals had already been infected for at least 79 days. This might explain the observation of waning anti-N antibodies, although further investigations with better defined samples are required before a definitive conclusion can be reached. 

This study has also highlighted the value of LIPS for the detection of antibodies in animals other than mink. Together with ferrets, the golden Syrian hamster is considered a valid animal model for COVID-19 [35,36], and so the presentation of a reliable serological assay applicable to these animal models that requires just one microliter of serum will be a valuable tool to better understand COVID-19 disease dynamics and antibody responses in this animal model. 

In conclusion, we believe that the characteristics of LIPS immunoassay and its ability to detect antibodies in multiple species make it an ideal candidate for the screening and identification of SARS-CoV-2 in multiple animal hosts. LIPS-S could represent a valid, biosafe and relatively facile serological assay to investigate circulation of SARS-CoV-2 in farmed, wild and laboratory animals, and could be used as part of an early warning system in disease control programs.

## Figures and Tables

**Table 1:** Description of the sera tested by LIPS.

Animal	Status	Number	Description	Confirmatory	Source/Ref
Mink	Positive	74	Infected farm animals	PRNT	IZSVe
	Negative	3	2 pet minks and 1 farmed mink	PRNT	IZSVe
Ferret	Positive	5	Experimental infection with SARS-CoV-2	ELISA, VNT	[24]
	Negative	8	Antibody negative, experimental animal	ELISA, VNT	FLI
Rabbit	Positive	1	Immunized with S1 peptide	ELISA, VNT	[25]
	Negative	2	Rabbit antisera to sheep (MP 55796)	ND	UVM
fruit Bat	Positive	2	Experimental infection with SARS-CoV-2	ELISA, VNT	[26]
	Negative	2	Antibody negative, experimental animal	ELISA, VNT	[26]
Hamster	Positive	22	Experimental infection with SARS-CoV-2	PRNT	IZSVe
	Negative	4	Non-infected, experimental animal	PRNT	IZSVe

IZSVe: Istituto Zooprofilattico Spermentale delle Venezie, Legnaro, Italy; FLI: Friedrich-Loeffler-Institut, Greifswald-Insel Riems, Germany; UVM: University of Veterinary Medicine, Vienna.

## Data Availability

The data presented in this study are available on request from the corresponding author.

## Supplementary Materials

The following are available online at https://www.mdpi.com/article/10.3390/v13081649/s1, Figure S1: Boxplot showing the differences in antibody levels between the negative and positive samples and the homogeneity of negative samples values across species for the LIPS-N assay, Table S1: Correlation (Spearman) between the LIPS-S assay, the LIPS-N assay and the NT according to time of sampling time, Table S2: Cohen kappa agreement between the LIPS-S assay, the LIPS-N and the NT according to time of sampling time.
