# Supplementary material for: Serological Detection of SARS-CoV-2 Antibodies in Naturally-Infected Mink and Other Experimentally-Infected Animals
Source: Viruses. 2021 Aug 19;13(8):1649. doi: 10.3390/v13081649 (PMC8402807; doi:10.3390/v13081649)
Supplement: Supplementary file 1 [file viruses-13-01649-s001.zip › Supplementary Table 2 Leg2.pdf]

Table S2. Cohen kappa agreement between the LIPS-S assay, the LIPS-N and the NT according to time of sampling time.

		Spike vs PRNT		Spike vs Nucleo		Nucleo vs PRNT	
	**Number of samples**	**Kappa**	**p-value**	**κ-value**	**p-value**	**κ-value**	**p-value**
All	77	1	0	0.05647	0.3008	0.05647	0.3008
Day 0	30	1	0	0.4483	0.03726	0.4483	0.03726
Day 7	25	1	0	0.3505	0.02912	0.3505	0.02912
Day 65	22	1	0	0.122	0.1551	0.122	0.1551

Table S2: Cohen kappa agreement between the LIPS-S assay, the LIPS-N and the NT according to time of sampling time.
