# Supplementary material for: Serological Detection of SARS-CoV-2 Antibodies in Naturally-Infected Mink and Other Experimentally-Infected Animals
Source: Viruses. 2021 Aug 19;13(8):1649. doi: 10.3390/v13081649 (PMC8402807; doi:10.3390/v13081649)
Supplement: Supplementary file 1 [file viruses-13-01649-s001.zip › Supplementary Figure 1 Leg2.pdf]

Figure S1

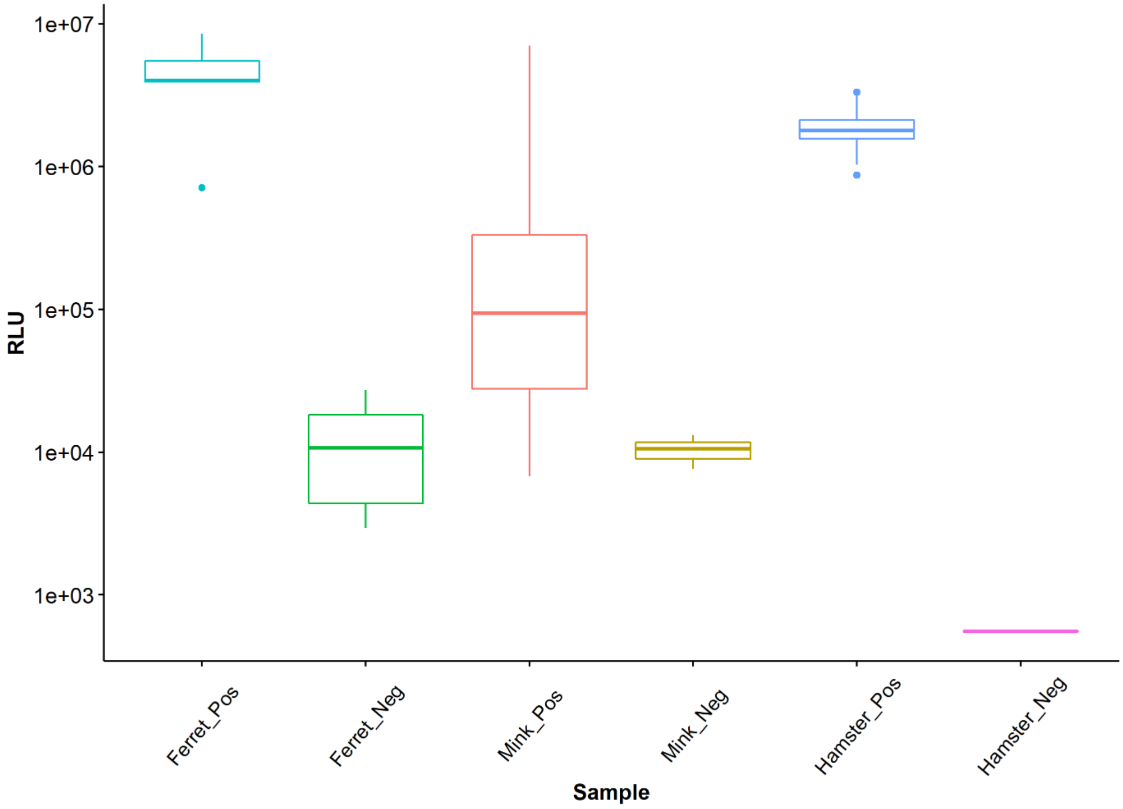

Figure S1: Boxplot showing the differences in antibody levels between the negative and positive samples and the homogeneity of negative samples values across species for the LIPS-N assay.
