# Supplementary material for: Serological Detection of SARS-CoV-2 Antibodies in Naturally-Infected Mink and Other Experimentally-Infected Animals
Source: Viruses. 2021 Aug 19;13(8):1649. doi: 10.3390/v13081649 (PMC8402807; doi:10.3390/v13081649)
Supplement: Supplementary file 1 [file viruses-13-01649-s001.zip › Supplementary Table 1 Leg2.pdf]

Table S1. Correlation (Spearman) between the LIPS-S assay, the LIPS-N assay and the NT according to time of sampling time.

		Spike vs PRNT		Spike vs Nucleo		Nucleo vs PRNT	
	**n**	**r**	**p-value**	**r**	**p-value**	**r**	**p-value**
All	77	0.7437793	9.248e-15	0.6831327	7.666e-12	0.6552078	1.011e-10
Day 0	30	0.7726795	5.636e-07	0.7543938	1.471e-06	0.675302	4.237e-05
Day 7	25	0.8661388	2.216e-08	0.8876923	3.284e-09	0.8914873	2.255e-09
Day 65	22	0.5056232	0.01637	0.3111237	0.1587	0.4911768	0.02027

Table S1: Correlation (Spearman) between the LIPS-S assay, the LIPS-N assay and the NT according to time of sampling time.
